# Interpreting results from oncology clinical trials: a comparison of denosumab to zoledronic acid for the prevention of skeletal-related events in cancer patients

**DOI:** 10.1007/s00520-012-1461-4

**Published:** 2012-04-27

**Authors:** George Dranitsaris, Eleftheria Hatzimichael

**Affiliations:** 1 Department of Haematology, University of Ioannina, Ioannina, Greece; 2 283 Danforth Avenue, Suite 448, Toronto, Ontario M4K 1N2 Canada

**Keywords:** Number needed to treat, Denosumab, Zoledronic acid, Breast, Prostate

## Abstract

**Background:**

Critically reviewing the design, endpoints, and results of clinical trials can be challenging to health care professionals. This paper will review the basic methods of presenting clinical outcomes in randomized trials and will focus on the number needed to treat (NNT) concept. NNT will then be applied to the case of bone-targeted therapies denosumab and zoledronic acid, which are used for the prevention of skeletal-related events (SREs) in a variety of disease sites.

**Methods:**

A Medline search was performed to identify randomized trials comparing denosumab to zoledronic acid for the prevention of SREs in patients with advanced breast, prostate, and other cancer sites. The data were extracted, and point estimates for the primary and secondary trial endpoints were converted into the NNT parameter.

**Results:**

NNT represents the number of patients that need to be treated with a new intervention in order to avoid one additional patient developing the event and is a powerful approach that can be used to make sense of numerical results from clinical trials. In patients with advanced breast, prostate, and other cancer sites, 18, 22, and 21 patients, respectively, would need to be treated with denosumab for at least 24 months to avoid one patient developing an SRE.

**Conclusions:**

The NNT approach is a simple and effective method to express the findings of randomized trials in a clinically meaningful way. In this analysis, the incremental benefits of denosumab would be realized when a minimum of 18 to 22 patients are treated for a prolonged duration. Clinicians would have to weigh the costs and benefits between denosumab and zoledronic acid when bone-targeted therapy is indicated.

## Introduction

Randomized controlled trials remain the gold standard for assessing the efficacy of new drugs. Yet, it is not always clear how to effectively interpret and use the trial findings. Health care decision-makers must choose methods to interpret data that are appropriate for the clinical question they wish to address. Various outcome measures such as event rates, relative risk (RR), relative risk reduction (RRR), absolute risk reduction (ARR), odds ratios (OR), and hazard ratios (HR) can be useful for understanding trial data from an epidemiological viewpoint and in setting expectations for aggregate clinical endpoints [20]. Such analyses may differ in scope between health policy issues, drug regulatory approval, or patient-specific clinical decisions. The current paper explains the number needed to treat (NNT) and number needed to harm (NNH) methods of analyses and discusses their virtues and potential pitfalls. The concepts are applied to a real-world example comparing two drugs, zoledronic acid and denosumab, both approved for the prevention of skeletal-related events (SREs) in various types of cancer patients.

### Does the new drug work better? Interpreting binary data

In clinical research, many outcomes are presented as binary (yes/no) and categorical outcomes. One of the most commonly used methods for analyzing binary data is the 2 × 2 contingency table (Table 1). Suppose that we are testing the ability of a new drug to prevent disease recurrence in high-risk cancer patients receiving adjuvant chemotherapy.

**Table 1:** The standard 2 × 2 contingency table

	Outcome		
Treatment	Event	No event	
Experimental	Cell_11_ = *a*	Cell_21_ = *b*	*n* = *a* + *b*
Control	Cell_12_ = *c*	Cell_22_ = *d*	*m* = *c* + *d*
			*N* = *a* + *b* + *c* + *d*

Two hundred patients who meet the study inclusion criteria are randomized into an experimental group where the new drug is given or into a control group where subjects receive the current treatment standard (100 subjects in each group). The primary objective of the trial is to compare the proportion of patients in each group who experience disease recurrence at 5 years. Let us assume that the results of the study reveal that 25 % of patients in the experimental group experience disease recurrence compared to 35 % in the control group.

Several methods can be used to describe these findings (Table 2). Some of the most commonly used measures of treatment effect include RR, RRR, ARR, and OR [9, 10, 12, 13]. Mathematically, RR is the incidence of an event in the experimental group divided by the incidence in the control group. In this example, the RR for disease recurrence with the new drug in the experimental group is ~0.71 (i.e., 0.25/0.35). This suggests that the risk of recurrence in the experimental group is lower compared to the control group. RRR is one minus the RR and is usually expressed as a percentage. In the chemotherapy example, the RRR for disease recurrence with the new drug is 1–0.71 or 29 % [9, 10, 12, 13]. Another very useful measure of benefit in binary data analysis is the ARR, which is the event rate in one group minus that in the other group (Table 2). In the current example, the ARR is 0.10 (i.e., 0.35 − 0.25).

**Table 2:** Binary clinical outcomes reported in randomized trials of adjuvant therapy in early stage cancers

Outcome	Definition	Expression
Early stage disease		
Disease recurrence	Number of patients who have redeveloped the disease at a given point in time, after a period of being disease-free.	Exp group = *a*
		Control group = *c*
Risk of recurrence	Proportion of patients who have disease recurrence divided by the number under investigation. Usually expressed as a percent.	Exp group = *a*/*n*
		Control group = *c*/*m*
RR	The risk of recurrence in the experimental group divided by the risk of recurrence in the control group. Usually expressed as a percent.	[(*a*/*n*)/(*c*/*m*)]
RRR	1-RR	1 − [(*a*/*n*)/(*c*/*m*)]
ARR	The risk of recurrence in the experimental group minus the risk of recurrence in the control group.	(*a*/*n*) − (*c*/*m*)
NNT	The number of patients that have to be treated by the new intervention in order to avoid one additional clinical event. In this case, a disease recurrence. Expressed as the reciprocal of the ARR (ARR^−1^).	[(*a*/*n*) − (*c*/*m*)]^−1^
OR	The odds of an event occurring in the experimental group divided by the odds of an event occurring in the control group.	(*a*/*b*)/(*c*/*d*)
HR	The instantaneous RR of an event per unit time for an individual in the experimental group compared with an individual in the control group, given that both individuals have survived to time *t*.	Ln[*h*(*t*)/*h*_0_(*t*) = *b*_1_ *x*_1_ + … *b*_*k*_ *x*_*k*_

### Number needed to treat

What makes the ARR useful is that if the reciprocal (ARR^−1^) is taken, one can estimate the number of patients that need to be treated with the new therapy to avoid one additional patient developing the event [9, 10, 12, 13]. To illustrate this point using the above example, 10 patients would need to be treated with the new chemotherapeutic agent to avoid one additional patient with disease recurrence (i.e., 1/0.10 = 10). However, if the new therapy was only 1 % better than the standard, 100 patients would need to be treated to derive the same benefit (i.e., 1/0.01 = 100). The value of the ARR and NNT becomes apparent in cost–benefit studies.

The advantage of NNT over other measures of clinical benefit is that it can easily be understood in terms of medical resource allocation by estimating how many patients would need to receive the new intervention in order to derive a unit of benefit [9, 10, 12, 13]. In order for the NNT to provide meaningful estimates, it must always be considered relative to a comparator group over a defined time period; NNTs cannot be estimated from a single-arm study. Also, it cannot be derived from trial endpoints that are on a continuous scale (e.g., patient blood pressure, cholesterol level, duration of relapse-free survival), as a time to event (e.g., time to first hospitalization, time to death), or as an RRR. In addition, if the experimental intervention is evaluated against a control group that is not the standard of care in a particular institution, then the NNT estimate would not be sufficiently meaningful.

### Number needed to harm

A closely related concept to the NNT is the NNH, which can be used to compare the safety profile of two treatments studied in a clinical trial [12]. As with the NNT, the NNH is treatment-specific and must always be relative to a comparator over a predefined time period. As an illustration, using the previous hypothetical example where the NNT with the new chemotherapy was 10, suppose that the absolute difference in the development of deep vein thrombosis was 10 % lower in the control group. For every 10 patients that we treat with the new agent, we avoid one patient with a disease recurrence (i.e., NNT = 10). However, we would also expect the development of one additional patient developing a deep vein thrombosis with the 10 treatments with the new therapy (i.e., NNH = 10). Clinicians must, therefore, consider both the NNT and NNH when interpreting the results of randomized trials and deciding on therapy for their patients.

### Limitations of NNT and NNH

NNT and NNH are both very useful for understanding the findings from randomized trials, but there are some caveats that must be taken into consideration [10, 12]. If the formulas are not used with sufficient rigor, the conclusions will be misleading. Here are some major pitfalls one may encounter when calculating and interpreting such analyses. NNT and NNH are disease-specific and comparisons across clinical conditions should not be performed. As an illustration, an NNT of 10 for preventing a breast cancer relapse is not comparable to an NNT of 5 for preventing febrile neutropenia with granulocyte colony-stimulating factor (G-CSF). It would be erroneous to conclude that G-CSF is preferred to the new chemotherapy that reduces breast cancer recurrences. The only situation where NNTs could be compared across studies is when the same disease condition is being investigated, when an identical control is used, and when the inclusion/exclusion criteria are comparable between trials. In addition, estimates should only be measured over the same time period. For example, it would be inappropriate to compare acute control of bone pain after a single fraction of radiation therapy to an oral bisphosphonate administered for 1 month because the time periods are not identical. Health care decision-makers should be wary of analyses that use unconventional measurements of time; NNT and NNH are very straightforward equations so results that are not reasonably intuitive should be viewed with caution.

Because NNTs and NNHs are presented as single numbers, the variability of treatment benefit may not be fully conveyed. Using confidence intervals (CI) is a way to account for this. From a practical point of view, the 95 % CI of the NNT and NNH can be calculated by taking the reciprocal of the 95 % CI limits of the difference in event rates between the experimental and control groups. For example, if the new chemotherapy agent reduces breast cancer recurrences by 10 % with the 95 % CI being between 8 and 12 %, the NNT would be 10 with a 95 % CI from 9 to 13 (i.e., 1/0.12 = 8.3 and 1/0.08 = 12.5; all NNTs should be rounded up to the nearest whole number).

One must also be aware that a calculated NNT represents the “average” for all patients who were enrolled into the randomized trial [10, 12]. This is helpful from an epidemiological point of view, but does not fully account for the potential differences in baseline disease risk between a clinician’s own patient population and those who were enrolled into the trial. Similarly, NNT assumes that a given RRR is identical across patients with different levels of risk. However, most of the benefit may be limited to the intermediate- and high-risk groups.

Subgroup analyses are very common in oncology clinical trials, but some of these analyses are not preplanned. Such unplanned analyses are very tempting to perform following a large trial because investigators are often interested in identifying the patient subgroups where benefit is maximized. However, performing multiple tests on a sample of data increases the risk of identifying statistically significant results by chance alone [8]. What is problematic in the clinical literature is that we have no idea how many statistical tests have been performed relative to the number that are presented in the published paper. Furthermore, a subgroup analysis only uses a portion of the original data and it is often underpowered to detect true differences between selected patient subpopulations. In order to minimize these potential biases, one should only estimate the NNT and NNH on the primary endpoint and in subgroups that were both preplanned and have a logical biological basis [9, 10, 12].

The final point to consider is that NNT and NNH analyses are often interpreted from a public health standpoint and can be used to guide medical resource allocation. Yet, NNT and NNH ignore the cost to treat each patient. Therefore, they are most useful when considered in a broader context.

### Bone-targeted therapies: application of NNT and NNH evaluations

NNT and NNH can be effectively applied to both health policy and clinical decision-making. We will use these concepts to compare the benefits of two agents, denosumab and zoledronic acid, both of which are approved for the prevention of SREs in patients with advanced breast, prostate, and other disease sites [2, 3, 11].

Bone metastases are common complications in advanced breast and prostate cancers occurring in approximately 70 and 14 % of patients, respectively [3, 17]. The SREs of clinical concern include hypercalcemia, pathologic fractures, spinal cord compression, radiation to bone, and surgery to bone [3]. The prevention of bony complications has been revolutionized by the use of bisphosphonates. When used in addition to systemic chemotherapy, bisphosphonates have significantly reduced and delayed the incidence of SREs. A meta-analysis of eight breast cancer trials showed that bisphosphonate use resulted in a 17 % relative reduction in the risk of developing an SRE [18]. The bisphosphonates currently in clinical use for the prevention of SREs include zoledronic acid, pamidronate, ibandronate, and clodronate [3].

The tumor produces various chemokines such as parathyroid hormone-related peptide, interleukin-8 (IL-8), and IL-1 to stimulate osteoblasts [21]. The osteoblasts induce the expression of the receptor activator of nuclear factor-kappa B ligand (RANK-L), which in turn induces osteoclast activity leading to increased bone resorption [14, 24]. Increased bone resorption causes the release of factors that favor the growth of malignant tumor cells [14, 24].

For many years, zoledronic acid has been the standard of care in multiple myeloma and patients with bone metastases from solid tumors including breast and prostate cancers, in conjunction with standard antineoplastic therapy [22, 23]. Denosumab is a monoclonal antibody that binds to RANK-L, thereby inhibiting its action [24]. It was recently shown via large randomized double-blind trials that denosumab significantly delays time to first SRE in prostate and breast cancers compared with zoledronic acid [7, 26]. In patients with other solid tumors and multiple myeloma, the drugs were shown to be equivalent in the median time to first SRE [11]. However, it should be pointed out that the mortality with denosumab was also higher than zoledronic acid in a subgroup analysis of patients with multiple myeloma. As a result, denosumab is not indicated for multiple myeloma [6].

## Application of NNT and NNH to zoledronic acid and denosumab

### Methods

A computer literature search of PubMed was conducted from January 2006 to January 2012 to identify randomized trials comparing denosumab to zoledronic acid for the prevention of SREs in patients with advanced breast, prostate, and other cancer sites. Search terms consisted of “{zoledronic acid}, AND {denosumab} AND {randomized clinical trial} AND {advanced breast cancer} OR {advanced prostate cancer} OR {cancer}.” The inclusion criteria for trial acceptance consisted of the following: published in a peer-reviewed English language journal, the trial must have utilized a parallel group randomized design, had to have been double-blinded, and the primary or secondary endpoint had to be the presentation of SREs defined as hypercalcemia, pathologic fractures, spinal cord compression, radiation to bone, and surgery to bone.

## Results

A randomized trial meeting the inclusion criteria was identified for the prevention of SREs in advanced breast and prostate cancers and in patients with other solid tumors or multiple myeloma [7, 11, 26]. The trials reported clinical and safety outcomes in terms of binary estimates, which was required for the NNT and NNH analysis.

### Breast cancer

In a study reported by Stopeck et al., denosumab was compared to zoledronic acid in 2,046 patients with advanced breast cancer. Eligible patients randomized one to one to either denosumab 120 mg subcutaneous injection or to intravenous injection of zoledronic acid 4 mg adjusted for creatinine clearance [26]. The study was a double-blinded non-inferiority trial and treatment was administered every 4 weeks. The primary endpoint was time to first SRE. The investigators reported that denosumab delayed time to first SRE (HR = 0.82, *p* = 0.01) relative to zoledronic acid [26]. Over the 34-month study duration, the proportion of denosumab and zoledronic acid patients developing at least one SRE was 30.7 and 36.5 %, respectively, for an absolute difference of 5.8 %. This absolute difference corresponds to an NNT of 18. Therefore, 18 patients would have to be treated with denosumab as an alternative to zoledronic acid for up to 34 months in order to avoid one patient developing an SRE (Table 3). To avoid a pathologic fracture and the need for radiation therapy to bone, approximately 39 and 27 patients would need to be treated with denosumab, respectively.

**Table 3:** NNT analysis for denosumab as an alternative to zoledronic acid

Endpoints	Dmab	ZA	Percent difference in event rate (ZA − Dmab)	NNT^a^
**Breast cancer**				
Sample size	1,026	1,020		
Any SRE	30.7 %	36.5 %	5.8 %	18
Type of SRE				
Bone surgery	1.2 %	0.8 %	−0.4 %	NA^b^
Cord compression	0.9 %	0.7 %	−0.2 %	NA^b^
Fracture	20.7 %	23.3 %	2.6 %	39
Radiation to bone	8.0 %	11.7 %	3.7 %	27
**Prostate cancer**				
Sample size	950	951		
Any SRE	35.9 %	40.6 %	4.7 %	22
Type of SRE				
Bone surgery	0.1 %	0.4 %	0.3 %	317
Cord compression	2.7 %	3.8 %	1.1 %	96
Fracture	14.4 %	15.0 %	0.6 %	163
Radiation to bone	18.6 %	21.3 %	2.7 %	37
**Other solid tumors and MM**				
Sample size	886	890		
Any SRE	31.4 %	36.3 %	4.9 %	21
Type of SRE				
Bone surgery	1.5 %	2.1 %	0.6 %	167
Cord compression	2.7 %	2.4 %	−0.3 %	NA^b^
Fracture	13.8 %	15.6 %	1.8 %	56
Radiation to bone	13.4 %	16.2 %	2.8 %	36

*Dmab* denosumab, *ZA* zoledronic acid, *MM* multiple myeloma, *NA* not applicable

^a^Reciprocal of the percent difference calculates the number of patients that need to be treated with denosumab in place of zoledronic acid in order to avoid one additional event. All NNT estimates were rounded to the nearest whole number

^b^An NNT could not be calculated because bone surgeries and cord compressions were at a higher incidence in patients treated with denosumab

Toxicity differences between the two drugs also need to be considered. This can be accomplished through the determination of the NNH. Fewer patients in the trial developed acute drug reactions and renal toxicity with denosumab (Table 4). In addition, there was a reduction in the number of treatment discontinuations due to adverse events in the denosumab group (9.6 vs. 12.3 %). In contrast, there were more patients developing osteonecrosis of the jaw (ONJ) and hypocalcemia. Therefore, the NNH for ONJ and hypocalcemia were 167 and 48 for denosumab, respectively (Table 4). In other words, for every 167 patients that are treated with denosumab instead of zoledronic acid, a clinician should expect up to four additional hypocalcemic episodes and one additional ONJ event.

**Table 4:** NNH analysis for denosumab as an alternative to zoledronic acid

Endpoints	Dmab	ZA	Percent difference in event rate (ZA − Dmab)	NNH^a^
**Breast cancer**				
Sample size	1,020	1,013		
Off drug due to adverse event	9.6 %	12.3 %	−2.7 %	NA^b^
Type of event				
ONJ	2.0 %	1.4 %	0.6 %	167
Hypocalcemia	5.5 %	3.4 %	2.1 %	48
Renal toxicity	4.9 %	8.5 %	−3.6 %	NA^b^
Acute reactions	10.4 %	27.3 %	−16.9 %	NA^b^
**Prostate cancer**				
Sample size	943	918		
Off drug due to adverse event	17.4 %	14.6 %	2.8 %	36
Type of event				
ONJ at 2 years	2.3 %	0.8 %	1.5 %	68
Hypocalcemia	12.8 %	5.8 %	7.0 %	15
Renal toxicity	14.7 %	16.1 %	−1.4 %	NA^b^
Acute reactions	8.4 %	17.8 %	−9.4 %	NA^b^
**Other solid tumors and MM**				
Sample size	878	878		
Off drug due to adverse event	10.4 %	12.4 %	−2.1 %	NA^b^
Type of event				
ONJ	1.1 %	1.3 %	−0.2 %	NA^b^
Hypocalcemia	10.8 %	5.8 %	5.0 %	5
Renal toxicity	8.3 %	10.9 %	−2.6 %	NA^b^
Acute reactions	6.9 %	14.5 %	−7.6 %	NA^b^

*Dmab* denosumab, *ZA* zoledronic acid, *MM* multiple myeloma, *ONJ* osteonecrosis of the jaw, *NA* not applicable

^a^Reciprical of the percent difference in side effect rates calculates the NNH. All NNH estimates were rounded to the nearest whole number

^b^An NNH could not be calculated because the event rates were at a lower incidence in patients treated with denosumab

In today’s climate of global economic recession and fiscal restraint, drugs’ costs also need to be taken into consideration. The acquisition cost of denosumab dose is approximately twice that of zoledronic acid in the USA. Combined with denosumab’s long duration of therapy to avoid one SRE, incremental drug cost may become a barrier to access, particularly in patients without drug insurance. To address the cost issue, one interesting editorial suggested that a cost-effective treatment strategy would be to offer zoledronic acid as a first-line therapy and then switch to denosumab using markers of bone turnover to identify patients who are most likely to benefit [27]. Notwithstanding, cost will remain a central question surrounding the selection of bone-targeting therapies.

### Prostate cancer

In the trial by Fizazi et al., which compared zoledronic acid to denosumab in prostate cancer, 1,901 patients meeting the eligibility criteria were randomized one to one to either denosumab 120 mg subcutaneous injection or to intravenous injection of zoledronic acid 4 mg adjusted for creatinine clearance [7]. The study was a double-blinded non-inferiority trial and treatment was administered every 4 weeks. The primary endpoint was time to first SRE. Patients randomized to receive denosumab had a longer time to first SRE (20.7 vs. 17.1 months; HR = 0.82, *p* = 0.008).

Over the 41-month study duration, the proportion of denosumab and zoledronic acid patients developing at least one SRE was 35.9 and 40.6 %, respectively, for an absolute difference of 4.7 %. This absolute difference corresponds to an NNT of 22. Therefore, 22 advanced-stage prostate cancer patients would have to be treated with denosumab as an alternative to zoledronic acid for up to 41 months to avoid one patient developing an SRE (Table 3). To avoid a patient needing bone surgery or radiation to bone, 317 and 37 patients would need to be treated, respectively. Similarly, to avoid a single patient developing a spinal cord compression or pathologic fracture, approximately 96 and 163 patients would need to be treated with denosumab, respectively (Table 3).

Denosumab was also safer than zoledronic acid in terms of acute drug reactions and renal toxicity. However, as was the case in breast cancer patients, there were more ONJ and hypocalcemic events with denosumab, with the respective NNH being 68 and 15 (Table 4). There was also an increase in drug discontinuation from adverse events in patients receiving treatment with denosumab. The NNH for treatment discontinuations with denosumab was estimated to be 36 (Table 4). In other words, for every 36 prostate cancer patients who start therapy with denosumab instead of zoledronic acid, one patient will have to stop therapy because of an adverse event.

### Other disease sites and multiple myeloma

Denosumab was also compared to zoledronic acid in patients with advanced solid tumors (excluding breast and prostate) as well as those with multiple myeloma [11]. The trial design and dosage of denosumab and zoledronic acid were identical to the former two trials. Overall, 1,776 patients were randomized to receive bone-targeted therapy over a 34-month trial horizon. The investigators reported equivalence in time to first SRE between drugs [12]. The proportion of patients developing any SRE was 31.4 and 36.3 % in the denosumab and zoledronic acid groups, respectively (the *p* value was not significant). The ARR of 4.9 % corresponded to an NNT of 21 (Table 3). Therefore, 21 patients would need to be treated with denosumab for up to 34 months in order to avoid one patient developing an SRE. Technically speaking, NNTs should not be determined for differences that are not statistically significant. However, they were applied to the trial results for comparative purposes.

When focusing on specific SREs, the NNT to avoid bone surgery, a pathologic fracture, and radiation to bone were 167, 56, and 36, respectively (Table 3). Patients treated with denosumab had a similar incidence of ONJ and a lower number of acute reactions, renal complications, as well as serious toxicities requiring discontinuation of therapy. However, the frequency of hypocalcemia was higher in the denosumab group, resulting in an NNH of 5 (Table 4).

## Discussion

The methods of NNT and NNH were described as a means of understanding trial results from an epidemiological viewpoint and setting expectations for clinical outcomes. To illustrate the methodology, these evaluations were applied towards bone-targeted therapies indicated for the prevention of SREs in advanced-stage cancer patients. The NNT to prevent one patient developing any SRE with denosumab in breast cancer, prostate cancer, and advanced solid tumors/multiple myeloma were 18, 22, and 21 over a 2- to 3- year time horizon. Therefore, clinicians and payers need to compare the benefits, risks, and the incremental drug cost of long-term therapy with denosumab. The trial data also suggest that oncologists can expect slightly more cases of ONJ and hypocalcemia with denosumab in patients with breast and prostate cancers [7, 26]. However, this would be compensated with fewer cases of acute drug reactions and renal toxicity.

The question of cost-effectiveness becomes important in the selection of bone-targeted therapy. The costs of treating an SRE have been estimated to be between $12,000 and $14,000 per patient [4, 14]. Therefore, denosumab may be a cost-effective alternative to zoledronic acid if it can avoid enough high-cost SREs. Recently, two economic evaluations evaluated the cost-effectiveness of denosumab in prostate and breast cancer patients. The first study used a Markov modeling approach to estimate the incremental cost per SRE avoided with denosumab in advanced-stage prostate cancer patients [28]. The study was conducted from the US payer perspective. The investigators estimated that the incremental total direct costs per SRE avoided with denosumab instead of zoledronic acid were $71,027 for 1 year and $51,319 for 3 years of therapy [28]. In a similar modeling analysis conducted in advanced-stage breast cancer patients, Carter et al. reported that it would cost $643,726 with denosumab to achieve one additional quality-adjusted life year over zoledronic acid [1]. Such an incremental cost-effectiveness ratio would be beyond the cost-effectiveness thresholds in most countries [5]. However, these two studies are in fact computer simulations of real-world clinical events. It waits to be seen with the collection of prospective observational data if denosumab is indeed able to avoid enough SREs to overcome the added drug cost.

It is of interest to note that an NNT analysis comparing denosumab to zoledronic acid in advanced solid tumors/multiple myeloma patients was also reported at the 2011 ASCO meeting [19]. The method used to calculate the NNT was unique and not consistent with the accepted approach [10, 12, 25]. In that analysis, the NNT for denosumab was calculated from the first SRE and again from the first and subsequent SREs. The NNT outcomes were then reported in terms of patient-years instead of individual patients. It was reported that the NNT for denosumab to prevent the first SRE event was approximately 9.9 and to prevent the subsequent SRE per year was 10. Upon closer inspection of their methodology, it appears that the investigators used the trial-reported RRR abstracted for the HR to calculate SREs. However, it is quite clear from the supporting literature that the ARR calculated from a binary trial endpoint is the appropriate parameter that should be used to calculate an NNT [10, 12, 25]. By definition, an NNT calculated from an RRR is not the number of patients that need to be treated to avoid one patient developing the event. As a result, the use of NNTs, as reported by Richardson et al., should be interpreted with caution [19]. Similar NNT analyses using this same methodology have also been done for the metastatic breast and prostate cancer studies comparing denosumab to zoledronic acid for the prevention of SREs [15, 16]. Similar caution should also be exercised in interpreting the NNT results from these studies.

There are a number of limitations in the current analysis that need to be acknowledged. The difference in the specific types of SREs avoided (e.g., fractures) was not statistically significant between the two drugs. Therefore, on technical grounds, the NNT to avoid each of these events with denosumab should not have been calculated because the true value would approach infinity, consistent with the lack of statistically significant differences in the subgroup analysis. Patient quality of life is an important component in studies evaluating bone-targeted therapies. However, the ability to prevent an SRE detected during a clinical trial does not necessarily correlate with quality of life improvements because many of these events may be asymptomatic. Consequently, NNTs are not able to capture differences in quality of life between the two competing therapies.

In conclusion, the NNT and NNH are effective ways of interpreting the data from randomized clinical trials. Both NNT and NNH are useful, but represent only one component in the overall decision-making process. Decision-makers must also consider drug safety, patient preferences, incremental drug cost, cost-effectiveness, and their own clinical judgment and treatment guidelines before offering a new agent to patients. Nonetheless, NNT and NNH analysis can make an important contribution towards the selection of optimal therapy for cancer patients, particularly in the case of bone-targeted therapies.
